# Metabolic Vulnerabilities in Multiple Myeloma

**DOI:** 10.3390/cancers14081905

**Published:** 2022-04-10

**Authors:** Julia S. L. Lim, Phyllis S. Y. Chong, Wee-Joo Chng

**Affiliations:** 1 Cancer Science Institute of Singapore, National University of Singapore, Singapore 117599, Singapore; csilslj@nus.edu.sg (J.S.L.L.); mdcsyp@nus.edu.sg (P.S.Y.C.); 2 Department of Medicine, Yong Loo Lin School of Medicine, National University of Singapore, Singapore 117599, Singapore; 3 Department of Haematology-Oncology, National University Cancer Institute of Singapore, National University Health System, Singapore 119074, Singapore

**Keywords:** multiple myeloma, metabolism, metabolic vulnerability

## Abstract

**Simple Summary:**

The advent of novel therapeutics has revolutionized the therapeutic scene of multiple myeloma (MM) and improved clinical outcomes significantly. Nonetheless, the disease remains incurable, especially in patients with refractory and relapsed disease. The emerging field of cancer metabolism has revealed metabolic vulnerabilities that can be exploited in myeloma. Altered glucose and glutamine metabolism are the most well-studied pathways in MM. In this review, we provide further insights into the scope of research that has been recently extended to these and other metabolic pathways and their implications for the disease and the tumor microenvironment. We also discuss some potential impacts of metabolism on myeloma prognosis and highlight mechanisms of drug resistance. Considering the challenges that abound, we deliberate on future knowledge gaps worth addressing.

**Abstract:**

Multiple myeloma (MM) remains an incurable malignancy with eventual emergence of refractory disease. Metabolic shifts, which ensure the availability of sufficient energy to support hyperproliferation of malignant cells, are a hallmark of cancer. Deregulated metabolic pathways have implications for the tumor microenvironment, immune cell function, prognostic significance in MM and anti-myeloma drug resistance. Herein, we summarize recent findings on metabolic abnormalities in MM and clinical implications driven by metabolism that may consequently inspire novel therapeutic interventions. We highlight some future perspectives on metabolism in MM and propose potential targets that might revolutionize the field.

## 1. Introduction

Multiple Myeloma (MM) is a plasma cell malignancy characterized by extensive heterogenous molecular and cytogenic subtypes, resulting in varied outcomes. It is the second most prevalent hematological malignancy globally [1]. High risk translocations (4;14), (14;16), including 17p13 deletion and 1q21 amplification, are associated with adverse outcomes and the endeavor to optimally manage these groups of patients remains elusive. The advent of novel drug classes such as proteasome inhibitors (PI), immunomodulatory drugs (IMIDs) and monoclonal antibodies have improved patient survival outcomes significantly. However, a long-standing clinical challenge remains, as patients eventually develop drug resistance represented by those with relapsed or refractory MM (RRMM) [2]. Hence, it is critical for novel strategies to be identified to enhance therapeutic interventions as MM remains incurable.

### Metabolic Deregulations Predict Adverse Prognosis in MM

Cancer cells adapt by reprogramming metabolic pathways, which is essential to ensuring that energy demands are met for rapid cell proliferation and tumor growth, across oxygen levels. Altered glucose and glutamine metabolism are the most well-studied pathways in MM, whereas serine metabolism and the pentose phosphate and folate pathways have also been implicated [3].

MM is a neoplasm with high prevalence in the elderly, with median age of diagnosis at 69 years [4]. The older population often present with parallel co-morbidities such as obesity, diabetes, and hyperlipidemia [5,6]. The novel association between metabolic syndrome (MS) and myeloma has recently been explored. Monoclonal proliferation of plasma cells within the bone marrow give rise to a secretion of paraproteins or M-proteins in the serum. The association of paraprotein production by myeloma cells with hyperlipidemia and low high-density lipoprotein (HDL) cholesterol has established a link between MM and features of MS [7]. Collectively, studies have found associations between MS features, inflammatory cytokines, and MM progression [8]. Moreover, some drugs indicated as treatment for metabolic disorders, including statins and metformin, could potentially improve outcomes in myeloma [9,10].

Furthermore, metabolic signatures could potentially influence prognosis in MM. Lactate dehydrogenase (LDH) is one of the prognostic factors that predicts for adverse outcomes in MM patients. A study reported a significantly reduced median overall survival in high-LDH patients compared to normal LDH levels (15 vs. 44 months, *p* 0.01) [11,12]. Accumulated evidence has shown that metabolic enzymes such as hexokinase II (HKII) and lactate dehydrogenase A (LDHA) are found in newly diagnosed myeloma patients, and their expression is further upregulated in relapsed MM patients, highlighting that elevated glucose metabolism is important in relapsed compared to newly diagnosed multiple myeloma [13,14]. Another study has recently used a validated prognostic model formulated based on the expression of seven genes involved in metabolic pathways. The authors employed a metabolic risk score formula which associated *CISH* with better survival outcome and the genes *NSDHL, CTPS1*, *FABP5, SLC25A5, FLNA* and *UBE2C* with poorer survival outcome. Patients classified in the high-risk metabolic group had significantly poorer survival rates (62% vs. 85%, *p* 0.001) compared to those belonging to the low-risk metabolic group [15].

Since metabolic rewiring is a hallmark of malignancy [16], coupled with its prognostic importance, it has generated significant interest in identifying metabolic pathways as therapeutic vulnerabilities in MM. In this review, we will discuss key metabolic abnormalities in MM, highlighting unique metabolic features and their gene signatures. Critically, insights into how metabolism drives clinical implications may birth potential therapeutic interventions. Here, we also postulate some future perspectives on metabolism in MM and propose potential targets that might revolutionize the field.

## 2. Current Literature of Metabolic Abnormalities in MM

### 2.1. Physiological Role of Metabolism in Plasma Cells

Long-lived plasma cells migrate and reside in the bone marrow to secrete antibody constitutively, thereby conferring lifelong protection [17]. Their specialized function of antibody secretion and limited replicative capacity demand specialized requirements on nutrient uptake and biomolecular synthesis. Plasma cell differentiation is initiated when naïve B cells are activated, and glucose uptake increases driving glycolysis and oxidative phosphorylation [18]. Single-cell transcriptomics of long-lived vs. short-lived plasma cells showed few differences, but they mainly differed in rates of glucose and amino acid uptake, which is significantly increased in long-lived plasma cells. It is reasonable to hypothesize that the determinant of plasma cells’ lifespan is attributed to cellular metabolism and not transcriptional regulation [19].

### 2.2. Myeloma Cells Undergo Metabolic Rewiring of Glycolysis and Mitochondria OXPHOS

Myeloma cells undergo extensive metabolic reprogramming, as is characteristic of all cancers [20]. Otto Warburg introduced the concept of the ‘Warburg effect’, referring to the hyper-elevation of glucose uptake by malignant cells. Aerobic glycolysis is a phenomenon where cancer cells metabolize glucose even in the presence of oxygen, while downregulating oxidative phosphorylation (OXPHOS). However, findings in conflict with this concept showed that many tumors had sufficient or even increased OXPHOS [21,22,23]. The dependency of MM cells on glucose has been shown to be evident by their sensitivity to multiple glycolytic inhibitors, including dichloroacetate [24]. Moreover, the expression of rate-limiting enzymes in the glycolysis pathway has been shown to be further upregulated with disease progression and to confer adverse prognosis [25]. Notably, MM cells can take up lactate exogenously through monocarboxylate transporter 1 (MCT1), thus fueling the reverse Warburg effect [26]. Notably, myeloma cells evidently metabolize using OXPHOS as a synergism between Metformin and ritonavir, an OXPHOS inhibitor and glucose uptake inhibitor, respectively, which induced apoptosis in MM cells. This suggests that the limitation of glycolysis is compensated with OXPHOS. Consistently, upon inhibition of glycolysis, glutamine dependency was demonstrated and, in this context, OXPHOS was mainly fueled by glutaminolysis [27]. Metabolic plasticity in MM cells is clearly highlighted, which presents it as a vulnerability to be targeted.

A steady level of acetyl coenzyme A (acetyl CoA) is required for maintaining the tricarboxylic acid (TCA) cycle. Therefore, the generation of acetyl CoA from multiple sources is critical, including its synthesis from citrate. Citrate is first transported outside the mitochondria by decarbonate antiporter solute carrier family 25 (SLC25A1), then it is reconverted into acetyl CoA and oxaloacetate by ATP citrate lyase (ACLY) in both the cytosol and nucleus [28,29]. Critically, a member of the decarbonate antiporter solute carrier family 25 was identified as a gene associated with poor survival outcome based on metabolic risk prognosis scores in MM [15]. Importantly, acetyl CoA was found to regulate chromatin modifications. It has an essential role in donating acetyl groups for acetylation, one of the key post-translational protein modifications. It can regulate chromatin dynamics and epigenetic control of gene expression by the activation of transcription machinery [30,31]. Histone acetyltransferases (HATs) are enzymes that catalyze the addition of acetyl groups at histone N-terminal tails. HATs are highly sensitive to alterations in acetyl CoA levels, while the latter is heavily dependent on glucose levels, fatty acid oxidation and mitochondria respiratory function [32,33,34].

### 2.3. Fatty Acid Metabolism Obesity as a Risk Factor in Myeloma

The role of bone marrow adipocytes (BMA) in supporting myeloma cells is relatively under-explored despite its dynamic functions. Its multifaceted roles include endocrine secretory functions, promoting cell-to-cell communication directly, correlating with obesity, a possible role in bone disease and close proximity to myeloma cells [35,36,37,38]. BMAs may potentially supply free fatty acids to MM cells for proliferation and survival. This has implications on fatty acid metabolism including fatty acid uptake and oxidation [39]. Myeloma cells have elevated levels of fatty acid-binding proteins (FABP), which potentially enhances tumor growth [40]. Furthermore, Etomoxir, an inhibitor of fatty acid beta oxidation and orlistat, an inhibitor of de novo fatty acid synthesis, ameliorated myeloma proliferation and decreased MM survival [41].t(4;14)-positive cells showed a high dependency on the mevalonate (MVA) pathway for survival. Inhibition of the fatty acid synthesis pathway with statin specifically increased apoptosis in this subset of cells. Furthermore, statin treatment led to an activation of the integrated stress response (ISR), which was modulated by co-administration with bortezomib. Evidence from exogenous rescue using geranylgeranyl pyrophosphate (GGPP) showed that t(4;14)-positive cells require the MVA pathway for the synthesis of geranylgeranyl pyrophosphate (GGPP). Interestingly, fluvastatin treatment had synergistic effects with bortezomib in vivo [42].

Obesity is a critical component of metabolic syndrome and contributes to MM pathogenesis heterogeneously. It is often measured based on body mass index (BMI) and classified into three unique stages by the World Health Organization: stage 1 (BMI 30–34.9), stage 2 (BMI 35–39.9) and stage 3 (BMI ≥ 40) [43]. Obesity-related epidemiological findings are deeply concerning and associations to multiple cancers including MM have been reported. Wallin and Larsson meta-analyzed 19 prospective studies which consistently demonstrated statistical significance between increased MM incidence and overweight individuals [44]. Indeed, excessive body weight has been highlighted as a critical risk factor for MM progression and mortality, which is well-supported by multiple studies [45,46]. Consequently, obesity has been established as a risk factor for MM by the International Agency for Research on Cancer recently [47]. It has also been proposed that the myeloma disease burden could be reduced at the population level with obesity accounted as the sole modifiable risk factor [8].

Several studies have found a positively correlated relationship between features of metabolic syndrome and MM, although the underlying mechanisms are not clear. Nonetheless, it has been postulated that adipose secretions of cytokines and proinflammatory mediators may be responsible for the stimulation of plasma cell activity and mitogenesis [43]. Critically, adipocytes secrete interleukin 6 (IL6), which is pertinent to the pathogenesis of MM and could possibly further increase lipid levels [8]. Additionally, insulin-like growth factor 1 (IGF-1) has been implicated in MM pathogenesis with its role of stimulating mitogenesis, promoting myeloma cell survival and secreting vascular endothelial growth factor, which is essential for angiogenesis [48]. Consistently, Metformin, an adenosine monophosphate (AMP)-activated protein kinase (AMPK) activator, was shown to have inhibitory effects on myeloma proliferation through the IGF-1R/PI3K/AKT/mTOR pathway and has synergistic effects with dexamethasone against MM [43].

Addressing obesity in MM is also imperative because adiponectin, which correlates inversely with body fat, is downregulated in MM and might account for progression from MGUS to MM [49]. A comparative study found a significantly increased MM risk which correlated with reduced adiponectin and resistin levels through bivariate analysis in a case control study [50]. Fowler et al. demonstrated direct evidence that reduced adiponectin levels correlated with MM progression through in vivo studies, as tumor burden and osteolytic bone disease increased in adipose-deficient mice with myeloma [37]. Adiponectin also confers protection by inducing apoptosis in myeloma cells through the stimulation of AMPK in line with tumor-suppressive effects of AMPK reported by other studies [51]. Furthermore, findings that adiponectin could suppress nuclear factor (NF)-κB activation and the production of interleukin 6 (IL6) and tumor necrosis factor alpha (TNF-α) [52] suggest that its downregulation may promote an increased secretion of these cytokines from the bone marrow [53]. Taken together, adiponectin presents as a novel target for MM and associated bone disease, and coupled with weight loss interventions may prevent MM progression, which warrants further investigation. A long-term administration of statins with lipid lowering properties may also confer protection from MM development, based on early subclinical disease, and may increase apoptotic levels in resistant MM cells. Despite the convincing evidence presented linking MM to metabolic syndrome, there is a lack of clinical trials studying the addition of cholesterol-lowering therapy or metformin in MM treatment in detail. 

### 2.4. PRL3: An Important Metabolic Regulator of Glycolysis and Serine/Glycine Metabolism

Phosphatase of regenerating liver-3 (PRL-3) is encoded by protein tyrosine phosphatase type IVA member 3 (*PTP4A3*) ([54], p. 3). It is an oncogenic phosphatase with dual-specificity [55] that is hyper elevated in multiple malignancies [56], including myeloma, and confers poor prognosis [57]. Importantly, in MM, PRL-3 is implicated in cytokine and growth factor signaling [58,59], and studies have validated its role in myeloma cell migration mediated by interleukin 6 [60]. Our lab has previously elucidated a positive autoregulatory feedforward loop in the IL6-STAT3-PRL-3 regulatory axis that can be therapeutically exploited. The pivotal role of the IL6 activation of STAT3 has implications for the elevated transcription of PRL-3, and an aberrant upregulation of PRL-3 expression leads to rephosphorylation of Signal Transducer and Activator of Transcription 3 (STAT3) when IL6 is absent. This mechanism drives prosurvival signals and mediates disease progression. Notably, an abrogation of PRL-3 reduced xenograft tumor growth and circumvented resistance to bortezomib (BTZ), which suggests that PRL-3 presents as a therapeutically amendable target in MM [61].

**Figure 1:**
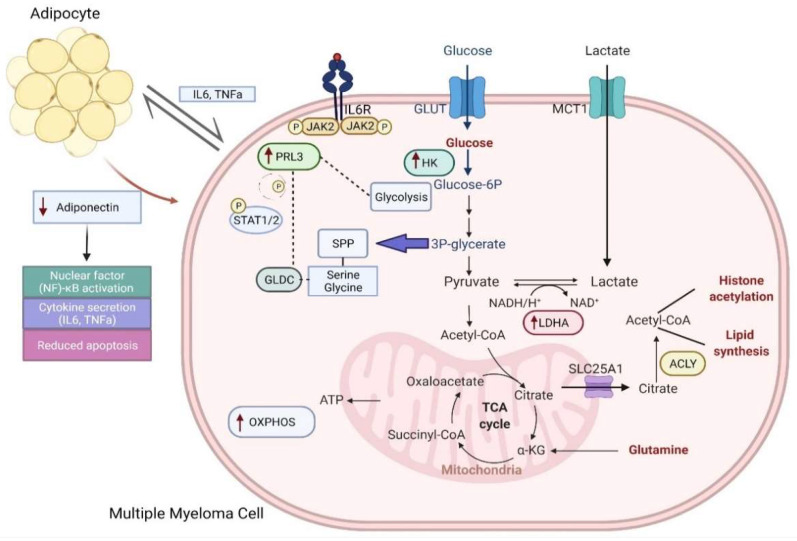
Schematic overview of critical metabolic alterations in myeloma and key proteins involved. Created with BioRender.com. [67].

A recent study provided evidence that adds PRL-3 as an important metabolic regulator in cancer. PRL-3 stimulation of glycolysis and other metabolic pathways did not increase proliferation, thus, it suggests that signaling molecules in the tumor microenvironment (TME) such as IL6 can cause metabolic shifts distinct from proliferative signals. PRL-3 upregulated aerobic glycolysis extensively in MM, as well as OXPHOS and, eventually, ATP levels. Glucose transporter 1 (GLUT1) and CD98, transporters for glucose and neutral amino acids, respectively, enabled the use of nutrients derived extracellularly [62]. Taken together, this resulted in glucose addiction evident by an increased vulnerability to a GLUT1 inhibitor, selective glucose transporter GLUT1 (STF31), which might be clinically exploited. Notably, PRL-3 increased the expression of glycolytic enzymes and those in the serine/glycine pathway. There was a positive correlation between *PTP4A3* and genes encoding for mitochondria serine/glycine enzymes. Previous reports have shown that glycine consumption and an increased expression of enzymes involved in the mitochondrial serine/glycine biosynthetic pathways are important metabolic adaptations in cancer cells [63]. Malignant cells mostly produce serine/glycine in the mitochondria, in contrast to healthy cells which produce these amino acids in the cytosol [64]. Glycine decarboxylase (GLDC) expression was most influenced by PRL-3 in MM. Its upregulation in cancer cells and its importance were previously validated in glioblastoma [65]. shRNA-mediated knockdown of GLDC and its activity in MM reduced cell viability significantly, possibly caused by the build-up of toxic metabolites from the conversion of accumulated glycine. Furthermore, knockdown of GLDC also reduced glycolysis in MM, which proves its role as a modulator of PRL-3-driven glycolysis. Consistently, Xu et al. demonstrated the role of PRL-3 in promoting glucose uptake and lactate export [66]. The study did not identify a specific underlying mechanism to explain the metabolic adaptation observed, but postulated that it was due to alterations in the expression or stability of important metabolic proteins. Interestingly, the PRL-3 modulation of metabolism was independent of hypoxia-inducible factor 1-alpha (HIF-1α), c-myc and AMPK based on immunoblots. An overview of the metabolic abnormalities in MM is schematically presented in Figure 1.

## 3. Clinical Implications of Metabolic Deregulation in Myeloma

Rewired metabolism attenuates the therapeutic effects of standard-of-care drugs, largely attributed to the hypoxic tumor microenvironment in the bone marrow (BM) [68]. Hypoxia-inducible factor (HIF)-1 is activated in this context, which drives glucose metabolism towards dependency on pyruvate conversion to lactate, rather than its oxidation in the mitochondria for energy production [25]. It has been postulated that drug resistance might arise through the adaptation to hypoxia in the BM, leading to relapse. Upregulation of HIF-1α and HIF-2α pathways was shown through an analysis of gene expression datasets comparing primary MM patients and healthy subjects. Importantly, a further enrichment of these pathways was evident in bortezomib-refractory and relapsed myeloma patients [13,69]. Human myeloma cell lines (HMCLs) subjected to hypoxic conditions and thereafter treated with bortezomib, dexamethasone and melphalan were observed to have increased glucose metabolism, with and overexpression of LDHA and HIF-1α post-treatment [25].

### 3.1. Implications of Resistance to Proteasome Inhibitors

Accumulating knowledge has generated novel therapeutics against glucose transporters in MM. Phloretin (GLUT1 inhibitor) in combination with the chemotherapeutic agent daunorubicin enhanced the latter’s effect in the hypoxic environment [70]. Besides GLUT1, inhibitors of GLUT4 such as compound 20 and ritonavir were found to sensitize MM cells with standard-of-care treatments [71]. Besides glucose transporters, hexokinases (HK) also present as a promising target. Under normoxic conditions, HK inhibitors 3BP, 2DG and lonidamine (LND) improved drug response in vitro, but no effect was seen in vivo [25,72,73]. In a hypoxic environment, bortezomib reduced the activity of HKII but not the activity of LDHA, suggesting a role of LDHA in BTZ resistance. Expectedly, upon LDHA knockdown, which reduced levels of lactate, bortezomib-resistant cells became sensitized to the drug. Consequently, enhanced mitochondrial activity, a reduced proliferation in hypoxia and thus a decrease in tumorigenicity was observed [25,74,75].

Proteasome inhibitors (PI) are extensively used as a treatment for MM [76]. PIs interfere with the unfolded protein response, but concurrently result in extensive metabolic changes evident through induced amino acid biosynthesis, antioxidant responses, lipogenesis and increased protein folding [77,78]. PIs present as a novel class of drug which targets cancer metabolism by affecting the homeostasis between protein synthesis, folding and destruction. Consequently, a rewiring of the MM metabolism may result in resistance to PI, as increased glycolysis or deregulated glucose metabolism have been found to contribute to bortezomib resistance using quantitative proteomic analysis [25,79]. Soriano et al. showed that PI resistance could be attributed to rewired metabolism and high oxidative phosphorylation [55]. Furthermore, in a pivotal study using mass spectrometry and whole metabolome profiling of PI-sensitive and resistant MM cells, resistant cells were characterized by global changes in metabolic pathways responsible for glutathione synthesis and regeneration, nicotinamide adenine dinucleotide phosphate (NADPH) and the TCA cycle. Collectively, this has functional consequences which improve antioxidant capacity and the preference for oxidative phosphorylation, resulting in more effective protein folding and reducing the proteasome load of misfolded proteins in PI-resistant cells [80,81]. PI-resistant cells were found to have mitochondria with structural changes and a change in lipid homeostasis [82]. Therefore, combination strategies targeting protein folding, energy supply, altered mitochondria metabolism or lipid homeostasis may be promising to overcome PI resistance.

Recent evidence has shown that bortezomib resistance could be attributed to serine metabolism. The serine synthesis pathway (SSP) is initiated either through extracellular input or by intracellular synthesis from glucose. It is noteworthy that the latter is the preferred pathway for biosynthesis in many cancers [83]. The SSP confers advantages to cancer cells and has implications in growth and proliferation [84,85]. Enzymes involved in the pathways, such as phosphoglycerate dehydrogenase (PHGDH), phosphoserine aminotransferase (PSAT) and phosphoserine phosphatase (PSPH), were observed to be upregulated in bortezomib-resistant and other HMCLs. Cells deprived of serine proved to improve bortezomib activity in RPMI-8226 and reduced tumor growth in mice with a deprivation of serine from diet [79,86]. This could be a promising way of treating MM and could potentially be applied as a diagnostic tool for PHGDH overexpression in tumorigenesis.

Additionally, drug resistance can be attributed to glutamine metabolism. Glutamine synthetase (GS) is minimally expressed in MM cells while Glutaminase (GLS) expression is heightened. Synergistic effects between the selective inhibitor CB-839 and proteasome inhibitors were observed both in vivo and in vitro [87].

As a proof of concept, protein folding capacity was compared between PI-sensitive and resistant MM cells tagged with mero-GFP. This enables quantitative real-time monitoring of functional protein folding dependent on the formation of disulphide bonds in the endoplasmic reticulum (ER) [88]. PI-resistant cells had an increased activity of protein folding together with increased disulphide bond formation. Chaperones required for protein folding are known to be highly dependent on ATP. Indeed, PI-resistant cells were found to have higher cumulative levels of ATP in the ER, consistent with ATP requirements for chaperone-driven protein folding. Critically, functional studies using protein disulphide isomerase inhibitor 16F16 and disulphide bond disrupting agent TCyDTDO showed synergistic effect on PI-adapted MM cell lines and patient-derived primary MM cells resistant to bortezomib and carfilzomib [89].

### 3.2. Implications on Melphalan Resistance

As a deoxyribonucleic acid (DNA)-alkylating agent, Melphalan is primarily administered as consolidation therapy together with ASCT (autologous stem cell transplantation) [90,91]. It remains clinically relevant as it has shown to improve progression-free survival in patients treated (50 months) relative to those untreated (36 months) [92].

Melphalan has similar properties to phenylalanine, an amino acid [93,94]. This mediates cellular uptake and transport into the nucleus, where DNA is intercalated, creating interstrand cross-links through an interaction with N7 of guanine or N3 of adenine [93,94]. DNA replication is inhibited by alkylating agents and cells are subjected to apoptotic cell death [95]. Despite advancements in the armamentarium of therapies against MM in recent years, resistance against alkylating agents has persisted, and an elucidation of mechanisms involved will prolong progression-free survival and improve patient outcomes. Koomen et al. demonstrated that metabolic alterations were associated with acquired Melphalan resistance using proteometabolomics [96]. Neither drug processing nor efflux were affected in the two resistant MM cell line models. Notably, transcriptomic evaluation of Melphalan resistance revealed an upregulation of genes involved in amino acid and glutathione (GSH) metabolism in addition to gene sets of the DNA repair and cell cycle [97,98]. Furthermore, higher concentrations of metabolites in the pentose phosphate pathway (PPP) and reduced guanine and guanosine levels were characteristic of resistant compared to sensitive cells. In line with melphalan’s mode of action, an increased efficiency in precursor formation and DNA synthesis through purine salvage prevents guanine bases from being covalently modified. Effective nucleotide synthesis coupled with efficient DNA repair contributes synergistically to melphalan resistance.

The pharmacological inhibition of 6-phosphogluconante dehydrogenase with 6-aminonicotinamide (6-AN) was able to circumvent melphalan resistance in both cell line models. It is worth considering the re-evaluation of drugs targeting purine metabolism, as perhaps the failed clinical trial of mycophenolate mofetil, an inosine monophosphate dehydrogenase inhibitor that did not improve patients’ outcomes significantly, was bypassed by other purine pathways such as hypoxanthine-guanine phosphoribosyl transferase and guanine monophosphate synthase [99,100]. Interestingly, purine metabolism and GSH metabolism were also modulated, but these were cell-line-specific, suggesting that resistance could be acquired by alterations of metabolic programs based on the baseline metabolism of each unique cell line. GSH has been linked to melphalan resistance [101] and data suggest that it may both neutralize the higher levels of ammonium cations and superoxide anions produced from increased purine synthesis and upregulate the expression in guanine deaminase and xanthine dehydrogenase/xanthine oxidase. The upregulation of GSH may arise due to oxidative stress and not specifically due to melphalan toxicity, and it could potentially have other roles in melphalan toxicity which remain to be determined.

### 3.3. Impact of Metabolism on Components of the BM Tumor Microenvironment

A bidirectional crosstalk between tumor cells and surrounding stromal cells in the BM microenvironment promotes drug resistance while creating an environment that favors tumor growth and the thriving of immune-suppressive cells [102,103,104]. Görgün et al. identified an elevated subpopulation of myeloid-derived suppressor cells (MDSCs) (CD11b^+^CD14⁻HLA-DR⁻/ˡᵒʷCD33^+^CD15^+^) with tumor-promoting and immune-suppressive activity in the peripheral blood and bone marrow of MM patients that is reduced in their healthy counterparts. Interestingly, the level of immunosuppressive cells identified increased with disease progression. A bidirectional interaction of MM cells with MDSCs has important consequences, as MM cells can mediate MDSC development and, in turn, MM cell growth is enhanced. Additionally, MDSCs have a suppressive effect on anti-tumor T cells such as CD8^+^ T cells and natural killer (NK) T cells. The administration of IMiDs and bortezomib in the microenvironment have proved effective against MM cells and CAM-DR in increasing median patient survival. However, a study demonstrated that these novel therapeutics could only modulate IL-6 and IL-10 cytokine expression. but not the immunosuppressive effect, and could not reduce the MDSC population [105].

Immunosuppressive cells such as MDSCs and regulatory T (Treg) cells thrive in acidic environments and aid in promoting tumor growth. Moreover, Treg cells rely predominantly on fatty acid oxidation rather than glycolysis, demonstrating the possibility of targeting specific cell populations. [106]. The “Reverse Warburg effect” is a phenomenon where myeloma cells release lactate in the microenvironment to activate oxidative phosphorylation in cancer-associated fibroblasts and drive secondary drug resistance mechanisms. As such, these cells were sensitized to Metformin-based combinations. Ideally, targeting metabolic vulnerabilities will have to take the microenvironment and immunosuppressive cells into consideration and concurrently aim to restore functions of anti-tumor immune cells. Additionally, targeting the interaction between MDSCs and MM cells may improve clinical outcomes and attenuate immunosuppression [105].

The interaction of MM cells with the bone marrow (BM) microenvironment is a basis for drug resistance in MM. Damiano and Dalton et al. conceived the term cell adhesion-mediated drug resistance (CAM-DR), which is the onset of drug resistance upon adhering to the extracellular matrix (ECM) [107]. Pyruvate kinase M2 (PKM2) was found to deregulate this mechanism in MM cell lines where its overexpression caused an attenuation of CAM-DR and knockdown of PKM2 enhanced the mechanism [108]. This is mediated by the regulation of PI3/AKT and MAPK/ERK1/2 signaling pathways involved in tumor progression [109]. Reelin, an extracellular matrix glycoprotein confers drug resistance by enhancement of glycolysis of HIF-1α [110].

### 3.4. Metabolic Deregulation Attenuates Immunotherapy

Immunotherapy is presently used for treatment in MM. The array of therapeutics used in MM includes immunomodulatory drugs (IMiDs), inhibitors of immune checkpoint, vaccines derived from dendritic cells and allogenic transplantation [111]. IMiDs potentiate the proliferative and functional properties of natural killer (NK) and NK T cells. Additionally, both daratumumab, a CD38 monoclonal antibody, and immune checkpoint inhibitors have shown to enhance T cell immunity against myeloma [112]. Other immunotherapeutic strategies include the dendritic cell (DC) vaccine synthesized by DC fusion with antigen and the chimeric antigen receptor (CAR) T cell therapy which modifies autologous T cells genetically with CAR expression and the specific target of tumor antigens [113].

Despite the promising potential of immunotherapeutic strategies, they come with their own set of challenges in the context of metabolism. Alterations in metabolism in the tumor microenvironment can weaken the therapeutic effect of immunotherapy [114]. The TME confers metabolic privileges to tumor cells by increasing the rate of glucose and glutamine uptake and by excessive lactate production and secretion. This metabolic shift is unfavorable for T cell recruitment and for them to thrive, because of nutrient deprivation, extensive acidification, a build-up of waste products and hypoxia [115]. Through pH buffering with bicarbonate, the acidification of the TME could be circumvented and the efficacy of immunotherapy improved. This could potentially be applied in MM. Although 2-Deoxy-d-glucose (2DG) is used in MM to inhibit glycolysis, it is incompatible with co-administration of immunotherapeutic agents as it impairs T cell metabolism and reduces its antitumor effects [114]. Immune cells primarily metabolize amino acid, such as L-arginine, which is a non-essential amino acid found in macrophages and DCs. However, lactate secretion by tumor cells leads to an overexpression of arginase, which converts L-arginine to urea and ornithine and, consequently, an impairment of T cell function by interference with cell cycle progression. MM cells are known to secrete lactate and it can be reasonably postulated that MM cells can cause T cell dysfunction through this mechanism [116].

#### 3.4.1. Crosstalk between Metabolic Rewiring and Lenalidomide Treatment

While cereblon (CRBN) is the primary target of IMiD anti-tumor activity, others have suggested the importance of myeloma metabolism in IMiD response [117]. A pivotal study found that CRBN plays a multi-faceted role beyond its ubiquitin ligase activity by binding to the lactate transporter monocarboxylate 1 (MCT1). IMiDs then execute their anti-tumor effect by competitively blocking this function of CRBN [118]. A follow-up study by the same authors further identified a complex network of transmembrane proteins and downstream candidate proteins, including L-type amino acid transporter 1 (LAT1 or SLC7A5)/CD98hc (SLC3A2). They highlighted LAT1/CD98hc, an amino acid transporter, as a metabolic vulnerability in MM upon IMiD treatment. This presents a promising therapeutic target for circumventing IMiD resistance, particularly in patients who overexpress LAT1/CD98hc [119].

A study assessed the effect of HIF-1 suppression on lenalidomide sensitivity in myeloma cells in vivo. Lenalidomide treatment downregulated HIF-1 minimally, did not modulate Ikaros Family Zinc Finger 1 (IKZF1) or Ikaros Family Zinc Finger 3 (IKZF3) expression, but downregulated Interferon Regulatory Factor 4 (IRF4) upon abrogation of HIF-1 α, suggesting IRF4 to be a target downstream of NF- kB downregulation. Consequently, lenalidomide-resistant MM cells were sensitized to lenalidomide treatment predominantly by inhibition of pathways involved in proliferation signals instead of anti-angiogenesis [120].

#### 3.4.2. Impact of Metabolic Alterations on the Regulation of T, and CAR-T Cell Functions through IMiDs

The efficacy of IMiDs also partly relies on the recruitment of immune cells, and lenalidomide was found to enhance CD8^+^ T cells, NK cells, Treg cells and MDSC cell populations [121]. Understandably, the presence of immunosuppressive cells in the MM microenvironment further complicates metabolic requirements. Notably, a subset of immune cells with anti-tumor activity, including CD8^+^ T cells, have overlapping metabolic properties with MM cells. This subjects them to inter-dependency and competition for metabolites with MM cells. Nonetheless, immune cells have less efficient mechanisms for acquiring nutrients compared to cancer cells and this results in an unfavorable microenvironment characterized by hypoxia and acidosis [122]. Notably, recent studies also highlighted the direct role of lipids in modulating T cell function, including infiltrating CD8^+^ T cells of myeloma. The lipid accumulation within T cells contributes to metabolic exhaustion mediated by lipotoxicity, and this is consistent with an upregulation of immune checkpoints such as Programmed cell death protein 1 (PD1) and 2B4 as well as an increase in immunosuppressive Treg cells. Importantly, the intracellular increase in cholesterol upregulates X-box binding protein-1 (XBP-1), a CD8^+^ T cell transcription factor. XBP-1 mediates the unfolded protein response and ER stress, which suppresses mitochondrial activity, elevates effectors of immune exhaustion and may therefore confound the anti-tumor activity of IMiDs [123]. XBP-1 can be targeted by STF-083010, an inhibitor of splicing, which proved effective in inducing cytotoxicity in MM cells and CD138^+^ cells in MM patients [124].

Lenalidomide is known to functionally modulate CAR T cells in MM by increasing its anti-tumor activity and persistence [125]. Metabolic profiling of CAR T cells with CD28 or 4-1BB signaling domains revealed plasticity in T cell metabolic reprogramming. The 4-1BB signaling domain promoted survival and correlated with an increase in the memory T cell population, mitochondrial biogenesis, and elevated oxidative phosphorylation, while antigen-stimulated CD28 CAR T cells mediated differentiation to effector T cells and resulted in increased glycolysis [126]. Downstream implications of metabolically programmed CAR T cells with effector or regulatory functions through these signaling domains could potentiate the effect of lenalidomide on CAR T-cell therapy.

#### 3.4.3. Impact of Adenosine Metabolic Alterations on Monoclonal CD38 Antibodies

Tumor microenvironments have an abundance of extracellular nucleosides that are metabolized by ectoenzymes for production of adenosine, which is known to regulate immune response. MM makes use of adenosinergic pathways to define its immune homeostasis. In this pathogenic context, adenosine acts as a local hormone by regulating cell metabolism based on purinergic receptors with varying affinities, which is expressed on immune, bone and tumor cells. This exploitation of metabolism leads to immunosuppression and mediates the impaired immune surveillance in cancer [127]. Consistently, plasma derived from myeloma aspirates contains high concentrations of adenosine, which increases with disease progression. It is also statistically relevant in the International Staging System for MM. CD38 is a protein with multiple functions, as both a receptor and ectoenzyme that is overexpressed at every stage in myeloma. If CD38 is active concurrently with CD203a and CD73 nucleotides, it catalyses the extracellular conversion of nicotinamide adenine dinucleotide (NAD^+^) to modulators of calcium signalling [128]. Adenosinergic activity is initialised after NAD^+^ is disassembled. Furthermore, cAMP released by tumor cells presents as a substrate for nucleotides to be metabolised to signalling adenosine [129].

Several studies have supported the view of considering immunometabolism to design original strategies against MM. An in-depth understanding of the metabolism of extracellular nucleotides will be useful for the development of therapeutics to inactivate adenosine-dependent immunosuppressive mechanisms. A resistant mechanism acquired against immune checkpoint inhibitors could be due to CD38-generated adenosine and thus, CD38-driven pathways that promote adenosine production may potentially be targeted therapeutically. Some strategies that are currently being evaluated are inhibiting nucleotide release channels; inhibiting adenosine production by blocking CD39/CD73, CD38/CD203a/CD73 ectoenzymatic pathways and employing drugs that can degrade extracellular adenosine [130,131].

Dysregulated metabolic environments may also weaken monoclonal antibodies (mAb) and decrease their therapeutic efficacy [132,133]. Some plausible explanations include fragmentation, aggregation, or denaturation together with a potential loss of mAb activity. Acidification of the TME affects the individual mAb’s properties and the environment where the mAb is required to exert its function [134]. Additionally, an acidic pH may cause aspartate, an amino acid, to degrade in the complementarity-determining regions (CDR), and therefore, the binding affinity between the mAb and its epitope may be weakened [132]. Collectively, acidosis in the TME of MM is highly relevant for determining the therapeutic efficacy of anti-CD38 mAbs. A summary of metabolism-associated resistance mechanisms and combination strategies with anti-myeloma agents is provided in Table 1.

## 4. Future Perspectives

### 4.1. Synergism between Dynamic Crosstalk of Epigenetics and Metabolism

Since dynamic crosstalk between epigenetics and metabolism exists, elucidating its important implications in cancer warrants further studies. Indeed, metabolic reprogramming could augment the available cofactors needed for epigenetic changes, oncometabolites may be agonists or antagonists for enzymes involved in epigenetics and they may affect epigenetic marks. Reciprocally, epigenetic deregulation can directly affect the expression of metabolic enzymes and affect the transduction of signals downstream which may regulate cell metabolism [135]. Accumulating evidence has led to the assumption of an active role of mitochondria in the determination of cell fate and function. Indeed, TCA cycle metabolites have been shown to regulate transcription factors and epigenetic modifications [34]. Genetic manipulation and pharmacological inhibitors can be employed to study the metabolic and epigenetic crosstalk in MM.

Rashid et al. used the quadratic phenotypic optimization platform (QPOP) to discover a novel and optimal drug combination of bortezomib with decitabine, an inhibitor of DNA methylase, in bortezomib-resistant MM cells [136]. DNA hypermethylation, a targetable signature of relapsed MM [137], was identified as a promising mechanism for inhibiting and restoring the expression of the tumor suppressor genes *CDKN1A* and *PTPN6*. It is unknown if there is a synergism between metabolism and epigenetics that enhances bortezomib resistance in MM. Epigenetic drug combinations have low success in being translated to clinics [138] and further studies to determine optimal drug doses, combinations and their dynamic crosstalk with metabolism may potentially advance the field.

### 4.2. c-MAF as a Regulator of OXPHOS

Patients with t(14;16) translocation have a deregulated c-MAF expression as the translocation subjects c-MAF to the influence of IgH enhancers. These patients constitute a high-risk group with adverse outcomes and a poor response to bortezomib [139], demanding in-depth scientific advances into the underlying molecular pathogenesis in order to unravel novel avenues of treatment. Critically, the occurrence of c-MAF overexpression in MM patients is ~50%, which is above the incidence of t (14;16) cases, with the t (4;14)-translocated subtype having the second highest c-MAF expression [140]. c-MAF confers innate resistance towards bortezomib, in which glycogen synthase kinase 3 (GSK3β) has been shown to regulate MAF protein stability and to ameliorate the activity of proteasome inhibition [139]. Since transcription factor-mediated phenotypes are not classical drug targets, elucidating pathways downstream or associated with c-MAF could present as potential therapeutic targets.

Critically, studies showed a class of recurrently deregulated metabolic genes in c-MAF signatures broadly classified with underexplored roles in MM [141,142]. Furthermore, c-MAF was found to be a critical molecular regulator of immune suppression in lung cancer by regulating macrophage metabolic reprogramming and effector function. It was found to control oxidative phosphorylation and the N-glycan synthesis pathway, thereby driving the macrophage phenotype. It was also found to regulate important enzymes in the TCA cycle and α-ketoglutarate (α-KG) levels and the uridine diphosphate N-acetylglucosamine (UDP-GlcNAc) pathway [143]. Notably, α-KG can be derived from fatty acid oxidation and epigenetic regulation [144], but mainly has glutaminolysis as its main source. Hence, this warrants further studies to gain deeper insights into deregulated metabolic pathways mediated by c-MAF. Consequently, functional studies of c-MAF-mediated metabolic vulnerability may be therapeutically exploited and emerge as a new therapeutic strategy for MM patients known to have a poor response to bortezomib. Taken together, metabolic perturbations may present as a likely key Achilles heel in MM and could potentially shift the current treatment paradigm 

## 5. Conclusions

Multiple myeloma is the second most common hematological malignancy, and the prognosis remains poor, with high incidences of relapse and mortality. Despite the advent of novel drugs, the disease is incurable, especially amongst elderly patients. One of the therapeutically challenging aspects of multiple myeloma treatment is the stratification of standard-risk vs. high-risk patients, as high-risk patients undergo rapid progression and therefore need more aggressive initial treatment and follow-up. For treatment advancement in multiple myeloma, it is critical to understand the underlying molecular abnormalities, which will improve risk stratification and better guide therapeutic decisions. Non-hyperdiploid myeloma cases are characterized by recurrent translocations that overexpresses a gene; this is concurrently its Achilles heel, as myeloma cells are subjected to oncogene addiction. Metabolomics holds immense potential for bench-to-bedside applications in therapeutics and diagnostics. Insights into metabolic pathways aberrantly activated in oncogenic-driven myeloma could be therapeutically exploited in each subset of patients. Moreover, employing the advanced technology of metabolomics, coupled with the ability to analyze large patient cohorts and with relatively simple sample preparation from blood, could signify a feasible application in clinics. Furthermore, targeting the metabolic phenotype of cancer cells is a strategic approach, as metabolomes are found downstream of transcriptome and proteome changes, and thus provide an accurate representation of cancer cell state. Further studies could potentially unveil novel drug combinations that could significantly improve patient survival in high-risk MM patients and overcome drug resistance. Collectively, we anticipate that future works will unravel novel treatment strategies and identify biomarkers to improve the overall prognosis and survival of MM patients.

## Figures and Tables

**Table 1:** Targets that can be co-administered with anti-myeloma agents to reduce resistance. HKII (Hexokinase II), OXPHOS (oxidative phosphorylation), SSP (serine synthesis pathway), GSH (glutathione), PPP (pentose phosphate pathway), 6-AN (6-Aminonicotinamide), TME (tumor microenvironment).

Anti-Myeloma Agent Mechanism of Action	Resistance Mechanism	Combination Treatment to Reduce Resistance
Bortezomib Proteasome inhibitor	Glucose transporters	Phloretin daunorubicin, compound 20 and ritonavir
	HKII	LDHA knockdown
	OXPHOS	Target mitochondria metabolism
	SSP	Serine starvation
	Glutaminolysis	CB-839
	Protein folding disulphide bond formation	16F16 TCyDTDO
Melphalan DNA alkylating agent	PPP	6-AN
	Reduced guanine and guanosine	Mycophenolate mofetil
	GSH	Reduce oxidative stress
Immunotherapy	Acidification of TME	Bicarbonate
	Adenosine	Degrade extracellular adenosine Inhibit adenosine production Inhibit nucleotide release channels

## Data Availability

Not applicable.
